# Nitrogen loss by anaerobic oxidation of ammonium in rice rhizosphere

**DOI:** 10.1038/ismej.2015.25

**Published:** 2015-02-17

**Authors:** San'an Nie, Hu Li, Xiaoru Yang, Zhaoji Zhang, Bosen Weng, Fuyi Huang, Gui-Bing Zhu, Yong-Guan Zhu

**Affiliations:** 1 Key Laboratory of Urban Environment and Health, Institute of Urban Environment, Chinese Academy of Sciences, Xiamen, China; 2 Key Laboratory of Drinking Water Science and Technology, Research Center for Eco-Environmental Sciences, Chinese Academy of Sciences, Beijing, China

## Abstract

Anaerobic oxidation of ammonium (anammox) is recognized as an important process for nitrogen (N) cycling, yet its role in agricultural ecosystems, which are intensively fertilized, remains unclear. In this study, we investigated the presence, activity, functional gene abundance and role of anammox bacteria in rhizosphere and non-rhizosphere paddy soils using catalyzed reporter deposition–fluorescence *in situ* hybridization, isotope-tracing technique, quantitative PCR assay and 16S rRNA gene clone libraries. Results showed that rhizosphere anammox contributed to 31–41% N_2_ production with activities of 0.33–0.64 nmol N_2_ g^−1^ soil h^−1^, whereas the non-rhizosphere anammox bacteria contributed to only 2–3% N_2_ production with lower activities of 0.08–0.26 nmol N_2_ g^−1^ soil h^−1^. Higher anammox bacterial cells were observed (0.75–1.4 × 10^7^ copies g^−1^ soil) in the rhizosphere, which were twofold higher compared with the non-rhizosphere soil (3.7–5.9 × 10^6^ copies g^−1^ soil). Phylogenetic analysis of the anammox bacterial 16S rRNA genes indicated that two genera of ‘*Candidatus* Kuenenia' and ‘*Candidatus* Brocadia' and the family of *Planctomycetaceae* were identified. We suggest the rhizosphere provides a favorable niche for anammox bacteria, which are important to N cycling, but were previously largely overlooked.

## Introduction

With the rapid increasing use of chemical fertilizers in agriculture, high nitrogen (N) loss in many densely populated countries is becoming an important issue from both environmental and agronomic perspectives. China is one of the largest rice producing countries and consumers of chemical N fertilizer in the world (FAO 2013, available at www.fao.org/publications/sofa.), which results in large amounts of N loss through NH_3_ volatilization, NO_3_^−^ runoff and leaching and N_2_O emissions (Xing and Zhu, 2000). However, >10% of the total N fertilizers applied on arable soils remain uncharacterized (Zhu, 2008).

The discovery of anaerobic oxidation of ammonium (anammox) in natural ecosystems provides new insights into the mechanisms responsible for N loss (Thamdrup and Dalsgaard, 2002; Dalsgaard *et al.*, 2003; Kuypers *et al.*, 2003). Recently, anammox bacteria were found to be widely distributed in agricultural fields (Humbert *et al.*, 2010; Wang *et al.*, 2012b; Shen *et al.*, 2013; Wang and Gu., 2013). However, these few published articles mainly focused on the distribution and phylogenetic diversity of anammox bacteria. The quantification of anammox activity and its contribution to the N cycle are not well known. To date, there are no studies that address anammox processes in the rhizosphere, which is ubiquitous (Jones and Hinsinger, 2008) and has a key role in N cycling (Richardson *et al.*, 2009; Jackson *et al.*, 2012; Liu *et al.*, 2014). The role of anammox bacteria in rhizospheric N cycling is unknown.

Hence, the objectives of the present study were to investigate the occurrence, activity, contributions and role of anammox to N loss in the rhizosphere and non-rhizosphere zones of a fertilized paddy soil from Southern China.

## Materials and methods

### Soil sampling

Paddy soil was collected from the Red Soil Ecological Experiment Station, Chinese Academy of Sciences, located in subtropical southern China, in Changde city, Hunan province (N: 28°57' E: 110°30'). Soil samples (0–20 cm) were collected in the field on 22 November 2010 and composited. Prior to the start of the experiment, the soil was air-dried, then homogenized and sieved (<2 mm).

### Experimental setup

Rhizo-bags (30-μm nylon mesh, 7.5 cm diameter, 12 cm height) filled with 475 g sieved soil were placed in the center of polyvinyl chloride pots (15 cm diameter, 23 cm height), which were then filled with 3 kg soil. The rhizo-bag was separated into two compartments, which allowed smaller molecular substrates to penetrate but prohibited penetration by roots (Supplementary Figure S1). Two treatments (control and N fertilization) were evaluated, and for each treatment, we sampled rhizosphere and non-rhizosphere soils. In the control treatment, no fertilizers were applied. In treated soil, N was applied as urea (100 mg kg^−1^ dry soil). The pot experiment was carried out using a randomized design with three replicates for each treatment.

The soils were first incubated for 1 month at 105% of water-holding capacity at 25 °C, then added to pots as described above. Rice seeds (cv. Xiangzaoshan 45) were sterilized in 30% H_2_O_2_ for 10 min and then thoroughly washed with de-ionized water. Three days after germination, uniform seedlings were transplanted into the rhizo-bags. After 37 days of rice growth in a greenhouse (illumination 1500 E m^−2^ s^−1^, ambient temperature 35 °C±2, 30 °C night; humidity, ambient 90%), rhizosphere and bulk soils were sampled. The rhizosphere and bulk soils were each divided into three parts. One part was used for *in situ* cell fixation for catalyzed reporter deposition–fluorescence *in situ* hybridization (CARD-FISH) as described below, another part was immediately frozen in liquid N_2_ and archived at −80 °C for molecular analysis, and the other part was incubated to determine anammox and denitrification activity.

### Chemical analytical procedures

Soil pH was determined in a 1:2.5 soil/water suspension. Soil organic matter, total organic C and total N were determined using a total carbon analyzer (TOC-V CPH, SHIMADZU, Japan). Ammonium (NH_4_^+^), nitrate (NO_3_^−^) and nitrite (NO_2_^−^) were extracted from the soil with 2 M KCl and diluted prior to determination by a flow injection analyzer (FIA QC8500, Lachat, Loveland, CO, USA). Soil grain size was analyzed using a laser scattering particle analyzer (MS2000, Malvern Instruments, Malvern, UK) after sieving (2 mm) to remove the gravel and plant roots. All analyses were performed in triplicate.

### Fluorescence *in situ* hybridization and catalyzed reporter deposition

CARD-FISH was applied to capture photographic documentation of anammox bacterial cells in the rhizosphere and bulk soils. The horseradish peroxidase (HRP)-labeled oligonucleotides probe Amx820 (5′-AAAACCCCTCTACTTAGTGCCC-3′) (Schmid *et al.*, 2000) was used for the detection of anammox bacteria, including ‘*Candidatus* Brocadia' and ‘*Candidatus* Kuenenia', in the soil samples. To check for unspecific staining and autofluorescence, the HRP-labeled probe Non338 (5′-ACTCCTACGGGAGGCAGC-3′) (Wallner *et al.*, 1993) was used. Total cells were determined on the basis of staining with DAPI (4,6-diamidino-2 phenylindole) and were recorded with an LSM 710 confocal laser scanning microscopy (Carl Zeiss, Inc., Oberkochen, Germany). Sampling and processing for CARD-FISH followed standard protocols (see Supplementary Table S1).

### Measuring anammox and denitrification rate with ^15^N labeled ammonium and nitrate

The activity and potential role of anammox and denitrification were measured at *in situ* soil temperatures with a ^15^N-tracing technique (Thamdrup and Dalsgaard, 2002; Risgaard-Petersen *et al.*, 2004). Approximately 3.5 g soil (fresh weight, three replicates) were transferred to 12.0 ml glass vials (Exetainer, Labco, High Wycombe, Buckinghamshire, UK) together with N_2_-purged media water from *in situ* irrigation water (total C: 0.89 mg l^−1^; total N: 0.40 mg l^−1^; NH_4_^+^-N: 0.08 mg l^−1^; NO_3_^−^-N: 0.29 mg l^−1^). The resulting paddy soils were then preincubated to remove residual NO_x_^−^ and oxygen (Supplementary Figure S2). Subsequently, 100 μl of N_2_-purged stock solution of each isotopic mixture, namely, (1) ^15^NH_4_^+^ (^15^N-(NH_4_)_2_SO_4_ at 99.14%, 12 mM N), (2) ^15^NH_4_^+^+^14^NO_3_^−^ (KNO_3_, 12 mM N) and (3) ^15^NO_3_^−^ (^15^N-KNO_3_ at 98.15%, 12 mM N), was injected through the septa of each vial, resulting in a final concentration of about 100 μM N. All isotope solutions were flushed with He prior to addition. The incubations were performed at temperature 35±1 °C. At five intervals over 24 h (0, 3, 6, 12, 24 h, respectively), reactions in three replicate vials from each treatment were inhibited by injecting 200 μl of a 7 M ZnCl_2_ solution. The rate and potential contribution to N_2_ formation by anammox or denitrification were calculated from the excess production of ^29^N_2_ and ^30^N_2_ in the ^15^NO_3_^−^ treatment, measured by continuous flow isotope ratio mass spectrometry (MAT253 with Gasbench II and autosampler (GC-PAL), Bremen, Thermo Electron Corporation, Finnigan, Germany), and the fraction of ^15^N in NO_3_^−^. The equations used and their explanations as described by Thamdrup and Dalsgaard (2002) are summarized in Supplementary Information (Supplementary Table S2).

### DNA extraction and PCR

DNA was extracted from approximately 0.25 g soil using a PowerSoil DNA Isolation Kit (MoBio Laboratories, Carlsbad, CA, USA), in accordance with the manufacturer's instructions. DNA concentration and quality were measured by a Nanodrop ND-1000 (Thermo Scientific, Wilmington, DE, USA). Thermal cycling and data analysis were carried out with a Real-time PCR Detection System (Roche480, Roche, Indianapolis, IN, USA) to assess the abundance of anammox *hzsA* gene with the primers of *hzs*A_1594F and *hzs*A_1857R (Kartal *et al.*, 2011; Harhangi *et al.*, 2012). The reaction mixture consisted of 4 μl DNA as template, 0.6 μl of each primer, 10 μl of SYBR 2 Premix Ex Taq, 1.0 μl BSA (20 mg ml^−1^) and 3.8 μl of dd H_2_O. Thermal cycling conditions were as follows: 3 min at 95 °C, followed by 45 cycles of 10 s at 95 °C, 10 s at 63 °C, 10 s at 72 °C, and 10 s at 82 °C. Three independent quantitative PCR assays were performed for each sample. Negative controls without DNA template were included in each amplification reaction. Standard curves were obtained using 10-fold dilutions of standard plasmid containing *hzs*A gene, which were amplified with the primers mentioned above. Every sample was quantified in three parallel quantitative PCR reactions to ensure the correct amplification. Only the reactions with efficiencies between 90% and 110% were accepted.

A nested PCR approach was conducted to detect the anammox bacterial 16S rRNA genes. The initial amplification was fulfilled using the PLA46f-630r primer combination with a thermal profile of 96 °C for 10 min, followed by 35 cycles of 60 s at 96 °C, 1 min at 56 °C, and 1 min at 72 °C (Juretschko *et al.*, 1998; Neef *et al.*, 1998). Afterward, a 500 times diluted PCR product was used as template for the second amplification with Amx368f - Amx820r primers using a thermal profile of 96 °C for 10 min, followed by 25 cycles of 30 s at 96 °C, 1 min at 58 °C, and 1 min at 72 °C. The PCR mixture and thermal cycling programs were conducted as described by Hefting *et al.* (2006). The amplified products were examined by electrophoresis using a 1.0% agarose gel.

### Cloning and sequencing

The PCR amplified anammox bacterial 16S rRNA gene fragments were cloned using the pMD19-T vector cloning kit (TaKaRa, Bio Inc., Shiga, Japan) according to the manufacturer's instructions. Plasmid DNA was isolated with the GeneJET Plasmid Miniprep Kit (Fermentas, Lithuania). At least 30 positive clones from each sample were randomly selected for sequencing (Invitrogen, Shanghai, China). The quality of the recovered sequences was checked using the Chromas Lite (version 2.01, Technelysium Pty, QLD, Australia) program. The occurrence of chimeric sequences was further examined using UCHIME (31). The Phylogenetic analysis of the 16S rRNA gene was performed with the MEGA 6.0 software (http://www.megasoftware.net) by maximum likelihood method. A bootstrap analysis with 1000 replicates was applied to estimate the confidence values of the tree nodes. The sequences obtained in this study for anammox bacteria are available from Genbank under accession numbers KJ523975–KJ524101.

### Statistical analysis

Results were given on a soil dry weight basis (oven dry, 24 h, 105 °C). Data were expressed as the mean of replicates±s.e. except where otherwise noted. One-way analysis of variance (Duncan, *P*<0.05) and Student's *t*-test (*P*<0.05) were used to determine differences between groups. All analyses were assessed by SPSS for Windows version 14.0 software (SPSS Inc., Chicago, IL, USA).

## Results

### Soil properties and inorganic N pools after planting

**Figure 1:**
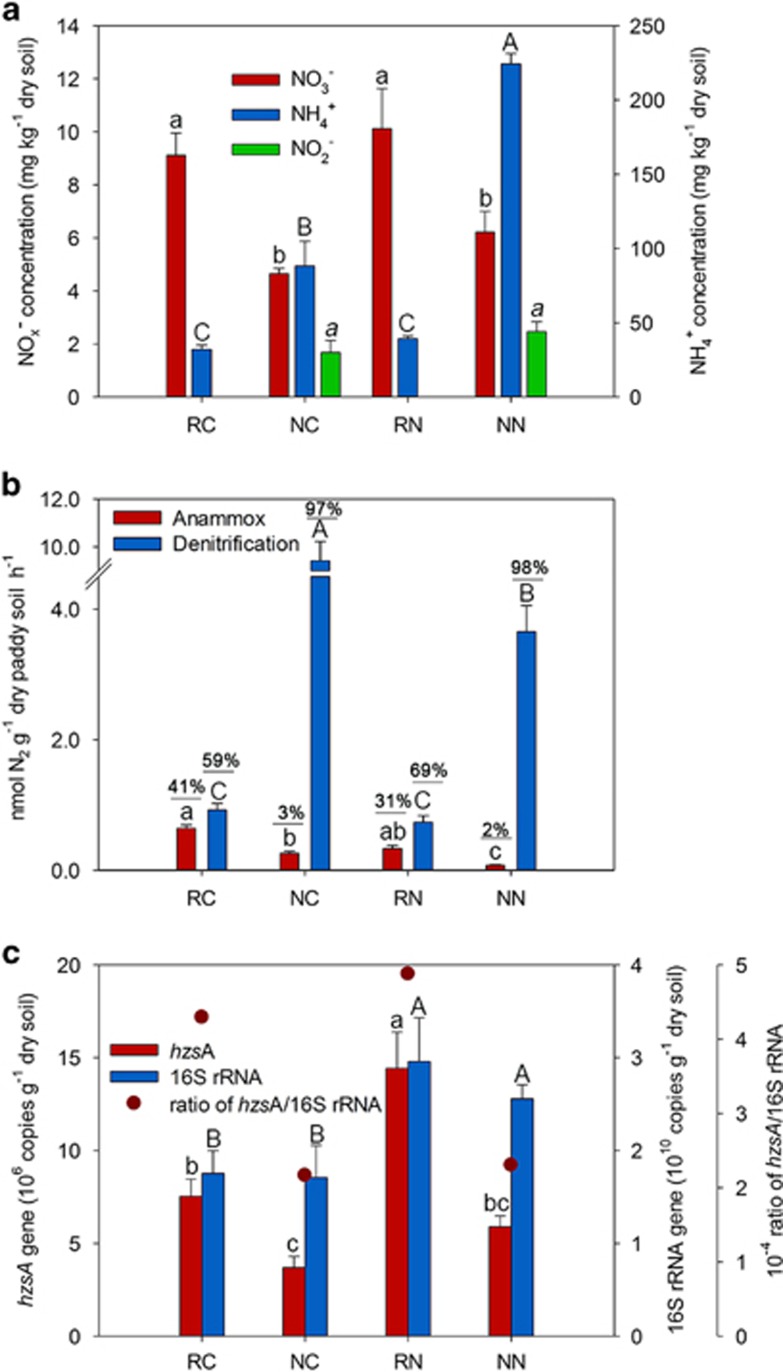
The concentration of NO_3_^−^, NO_2_^−^ and NH_4_^+^ (**a**), anammox, denitrification activity and their contributions to total N_2_ production (**b**) and abundance of anammox bacteria targeting the *hzs*A gene and total bacteria targeting the 16S rRNA gene (**c**) both in the rhizosphere and non-rhizosphere soils. The soil samples evaluated were (1) rhizosphere in control (RC); (2) non-rhizosphere in control (NC); (3) rhizosphere in N fertilization (RN); and (4) non-rhizosphere in N fertilization (NN). *n*=3, Duncan test or *t*-test at *P*<0.05 level, letters with different labels indicate significant differences.

Paddy soil was selected for this study with high ammonia concentration, which was also used in many anammox bioreactor studies to stimulate anammox bacteria (Strous *et al.*, 1998; Sliekers *et al.*, 2002; Kartal *et al.*, 2008). The chemical characteristics of the soil are shown in Table 1. Because of long-term application of chemical fertilizers, the content of paddy soil organic matter, C_mic_, N compounds (total N, N_mic_) and available P and K were high. The pools of NH_4_^+^ and NO_x_^−^ from planted soils are shown in Figure 1a. Overall, the NH_4_^+^ concentrations were almost 1–2 orders of magnitude higher than those of NO_x_^−^. The NH_4_^+^ concentrations in the bulk soil were significantly higher than in the rhizosphere. In the rhizosphere, the NO_3_^−^ concentrations were significantly higher than in the bulk soil. The NO_2_^−^ concentration in the non-rhizosphere soil ranged from 1.7 mg kg^−1^ to 2.8 mg kg^−1^ but was undetected in the rhizosphere soil. The NH_4_^+^ and NO_3_^−^ concentrations in the rhizosphere did not change significantly between fertilized and non-fertilized soil.

### Detection of anammox bacterial cells

**Figure 2:**
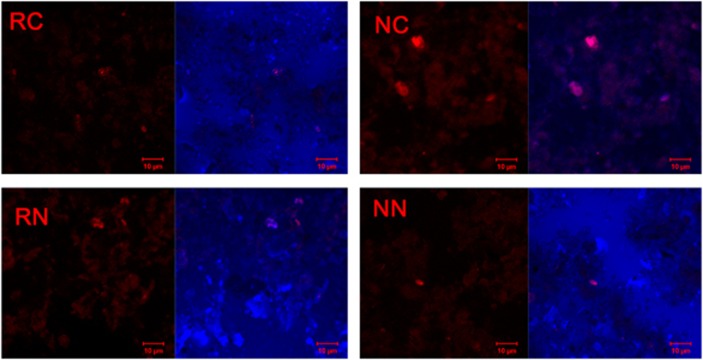
*In situ* mapping of anammox bacteria from rhizosphere and bulk soils by confocal laser scanning confocal microscopy. Anammox bacteria stained by CARD-FISH probe Amx820 specific for genera ‘*Candidatus* Brocadia' and ‘*Candidatus* Kuenenia' (left) and combination of DAPI-stained cells and cells stained with probes specific for anammox bacteria (right) are shown. NC, non-rhizosphere in control; NN, non-rhizosphere in N fertilization; RC, rhizosphere in control; RN, rhizosphere in N fertilization.

For the analysis of anammox bacterial cells in flooded rice soils, CARD-FISH analysis was used, which allowed for the localization of native microbial cells in the rhizosphere and bulk soils *in situ*. Probe Amx820 hybridized with their 16S rRNA was constructed to specifically detect anammox organisms, including ‘*Ca*. Brocadia' or ‘*Ca*. Kuenenia'. The acquisition of CARD-FISH signals and autofluorescence were performed by laser scanning confocal microscopy for an improved visualization of microbial cells. Images showing discrete fluorescent signals of high intensity were observed, which represented individual microbial cells in the soils (Figure 2). Anammox bacteria, including genera affiliated with ‘*Candidatus* Brocadia' and/or ‘*Candidatus* Kuenenia,' were detected in samples from both the oxic and anoxic zones and verified by CARD-FISH and sequencing of their 16S rRNA genes.

### Anammox and denitrification rate and contribution to N_2_ production

To determine anammox rate and the potential role of anammox as a N_2_ producer, incubations were performed with rhizosphere soil and bulk soil under *in situ* temperature using a ^15^N isotope-tracing technique. The results showed that, in the soil samples amended with ^15^NH_4_^+^, no significant accumulation of ^15^N_2_-labeled gas could be detected in the rhizosphere or bulk soils (Supplementary Figure S3A), indicating that all ambient ^14^NO_x_^−^ was consumed during preincubation. When both ^15^NH_4_^+^ and ^14^NO_3_^−^ were added, ^29^N_2_ was accumulated but not ^30^N_2_ (Supplementary Figure S3B). Significant rates of both anammox and denitrification were observed in the incubations amended with ^15^NO_3_^−^ only (Supplementary Figure S3C). It is possible that potential rates may overestimate the actual *in situ* activity because NH_4_^+^ and NO_3_^−^ availability may limit the process *in situ*. However, the *in situ* concentration of NH_4_^+^ and NO_3_^−^ were quite high (32.0–224.3 mg kg^−1^ and 4.6–10.1 mg kg^−1^, respectively). Our samples were incubated with NH_4_^+^ or NO_3_^−^ to a final concentration corresponding to a maximum of <10% of the *in situ* concentration, therefore the potential rates of anammox and denitrification may not be seriously overestimated.

Anammox and denitrification rates calculated using ^29^N_2_ and ^30^N_2_ production values from the ^15^NO_3_^−^ incubations are shown in Figure 1b. High potential rates of anammox were observed (0.33–0.64 nmol N_2_ g^−1^ soil h^−1^) in the rice rhizosphere for both treatments, contributing 31% and 41%, respectively, to their total N_2_ loss. In the non-rhizosphere zone, however, the rates of denitrification (3.66–9.42 nmol N_2_ g^−1^ soil h^−1^) were much higher than that of anammox (0.08–0.26 nmol N_2_ g^−1^ soil h^−1^). Approximately 2–3% was produced by anammox, while the remainder was denitrified to N_2_. Though differences were observed in denitrification activity between rhizosphere (0.74–0.92 nmol N_2_ g^−1^ h^−1^) and non-rhizosphere (3.66–9.42 nmol N_2_ g^−1^ h^−1^), denitrification was still the main contributor of N_2_ production in paddy soil (59–69% in rhizosphere and 97–98% in non-rhizosphere). The isotope tracing technique revealed that the ratio of contribution to N_2_ production by anammox in rhizosphere (31–41%) was mostly 15 times higher than that in non-rhizosphere (2–3%), suggesting high variability in N loss between the two zones in the paddy soil.

### Abundance of anammox bacteria

To obtain more detailed information on the anammox bacteria, the primer pair of *hzs*A_1594F and *hzs*A_1857R was applied for the quantification of anammox bacterial *hzs*A gene abundance in the soil using quantitative PCR assay (Harhangi *et al.*, 2012; Wang *et al.*, 2012b). The *hzs*A gene abundance was up to 0.75–1.4 × 10^7^ copies g^−1^ dry soil in the rhizosphere, whereas the number of anammox bacterial genes decreased to 3.7–5.9 × 10^6^ copies g^−1^ dry soil in the non-rhizosphere (Figure 1c). The quantitative PCR assays on 16S rRNA gene was also performed and showed the proportion of anammox cell numbers to total bacteria were maintained at a high level of 4.3–4.9 × 10^−4^ in the rhizosphere in comparison to non-rhizosphere (2.2–2.3 × 10^−4^). Anammox bacterial abundance and their proportion to total bacteria detected in rhizosphere were about twofold higher than those observed in the adjacent bulk soils.

### Community structure of anammox bacteria

**Figure 3:**
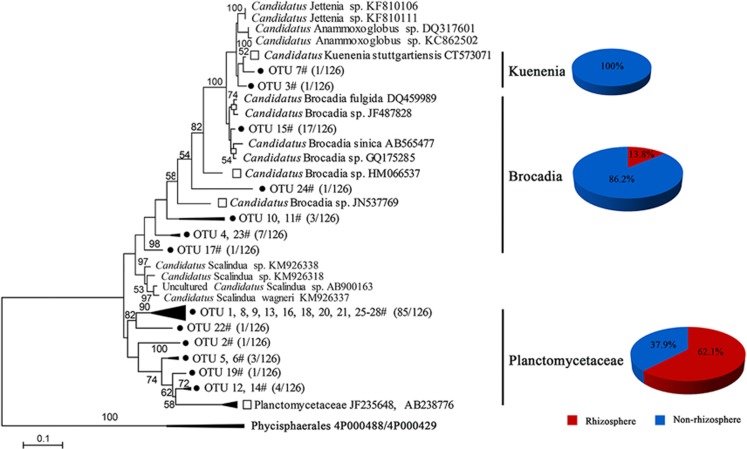
Phylogenetic tree of deduced anammox 16S rRNA gene sequences. Branches corresponding to partitions reproduced in<50% bootstrap replicates were collapsed.

To investigate the community structure of anammox bacteria, the anammox 16S rRNA gene clone libraries were constructed from four representative soil samples (RC, NC, RN and NN). A total of 28 operational taxonomic units (OTUs) (97% cutoff) were obtained from the rhizosphere (11 OTUs from RC and 9 OTUs from RN) and the non-rhizosphere (11 OTUs from NC and 8 OTUs from NN) as shown in Figure 3. Phylogenetic analyses of anammox 16S rRNA bacterial sequences and related sequences deposited in GenBank showed 19 OTUs were assigned to *Planctomycetaceae* (rhizosphere, 62.1% non-rhizosphere, 37.9%). The OTU 3# and OTU 7# in the non-rhizosphere were most closely affiliated to ‘*Candidatus* Kuenenia'. The remaining OTUs were affiliated with ‘*Candidatus* Brocadia' (rhizosphere, 13.8% non-rhizosphere, 86.2%).

## Discussion

To the best of our knowledge, this is the first report of the abundance and activity of anammox bacterial in rice rhizosphere. Up to 31–41% of rhizospheric soil N_2_ production with rates of 0.33–0.64 nmol N_2_ g^−1^ soil h^−1^ was contributed by anammox, whereas approximately 2–3% of N was produced through anammox in bulk soils (0.08–0.26 nmol N_2_ g^−1^ soil h^−1^). These findings improve our understanding of N cycle in paddy fields.

The observed anammox rates (0.33–0.64 nmol N_2_ g^−1^ h^−1^) in the rhizosphere were significantly higher than that in the bulk soils (0.08–0.26 nmol N_2_ g^−1^ h^−1^), indicating that rhizospheric anammox process might be an overlooked pathway for N loss from paddy soils. In the rhizosphere, which is exposed to oxygen, both partial denitrification and nitrification may produce nitrite for anammox bacteria, which have been reported in many environments, including marine (Kuypers *et al.*, 2005; Lam *et al.*, 2007), freshwater (Zhu *et al.*, 2010, 2011a; Wang *et al.*, 2012a) and soil (Zhu *et al.*, 2011b). In oxygen-limited bulk soil, where nitrification is inhibited, denitrification may provide anammox with nitrite. Higher anammox rates were also detected in the surface layer of other paddy soils (Zhu *et al.*, 2011b; Sato *et al.*, 2012). The rhizosphere and surface layer of standing water are typical oxic–anoxic interfaces in wetland ecosystems; therefore, we propose that the redox gradient in the rhizosphere is a hotspot for anammox activity. In waterlogged ecosystems, oxygen exposure and NO_x_^−^ production could be the key factors in determining anammox activity.

In the present study, great variation in denitrification rate was observed between rhizosphere (0.74–0.92 nmol g^−1^ soil h^−1^) and bulk soils (3.66–9.42 nmol N_2_ g^−1^ soil h^−1^) (Figure 1b). As high concentrations of nitrate were detected in both zones, the possible reason for these changes may be the presence of oxygen, which was transported through the roots (Armstrong, 1971; Brune *et al.*, 2000). It is known that denitrification activity declines sharply in the presence of oxygen (Firestone *et al.*, 1979; Firestone and Tiedje., 1979). Hence, we propose that due to oxygen exposure denitrification activity in the rhizosphere declined significantly in comparison to the adjacent anoxic bulk soil. Although there are differences in denitrification rates between the rhizosphere and bulk soils, our results indicate that denitrification is still the main pathway for N loss in paddy soil (59–69% in the rhizosphere and 97–98% in the non-rhizosphere).

In addition to their high activity, high cell numbers of anammox bacteria (0.75–1.4 × 10^7^ copies g^−1^ soil) in the rhizosphere in comparison to non-rhizosphere were also observed and were higher than those reported in other agricultural soils (Zhu *et al.*, 2011b; Humbert *et al.*, 2012; Shen *et al.*, 2013). To the best of our knowledge, this is the highest anammox abundance recorded in agricultural soils. In the rhizosphere, the abundance was over twofold higher compared with the non-rhizosphere zone. The reason for the drastic increase in abundance was probably related to NO_x_^−^ in the rhizosphere, which was in agreement with other studies (Hamersley *et al.*, 2007; Shen *et al.*, 2013; Zhu *et al.*, 2013). Another possible reason may be attributed to the high background value of NH_4_^+^ (32.0–224.3 mg kg^−1^) in comparison to NO_3_^−^ (4.6–10.1 mg kg^−1^). In the pot experiment, no differences in rhizospheric NH_4_^+^ concentrations were observed between fertilized and non-fertilized treatments, which was in agreement with literature reporting that 10 units of anammox reaction needed 10 units of ammonia and nitrate, respectively (Jetten *et al.*, 1998; Zhu *et al.*, 2013). This suggests that the rhizosphere provides a more favorable habitat for anammox bacteria.

In the present study, the anammox communities were closely related to two genera ‘*Candidatus* Brocadia' and ‘*Candidatus* Kuenenia', which have been observed to be dominant anammox community in soils and sediments (Fan *et al.*, 2010; Humbert *et al.*, 2010; Zhu *et al.*, 2011a; 2011b; 2013; Shen *et al.*, 2013). Moreover, results using a 16S rRNA-targeted oligonucleotide probe specific for these two genera demonstrated the actual presence of anammox bacteria (Figure 2) in rhizosphere and bulk soils. The Amx820 oligonucleotide probe was one of the most widely used probes for the CARD-FISH analysis (Schmid *et al.*, 2005; Li and Gu, 2011). It was the first time that CARD-FISH was applied to detect anammox bacterial cells in flooded rice soils.

**Figure 4:**
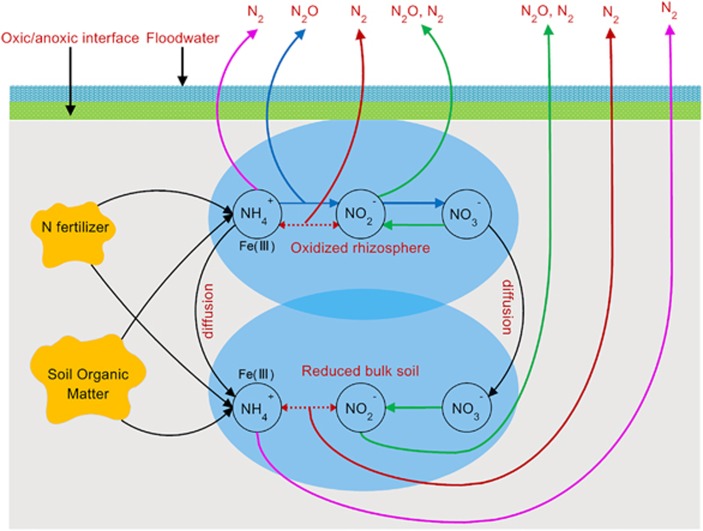
Schematic representation of the N loss from paddy soil. The classical processes of nitrification (blue), denitrification (green) and recently discovered anammox (red) as well as anaerobic oxidation of ammonium coupled to Fe-reduction (magenta) are shown both in oxidized rhizosphere and reduced bulk soil.

It should also be noted that the rhizosphere is an operational definition, which is dependent on experimental setup. In this study, we separated bulk soil from rhizosphere using a nylon bag, which was commonly adopted in other rhizosphere studies (Steen, 1984; Steen and Atkinson, 1991; Liu *et al.*, 2006; Jia *et al.*, 2013; Huang *et al.*, 2014). Nonetheless, in reality the micro-environment around rhizosphere is a continual redox gradient that extends from the root surface to the bulk soil. This redox gradient is common for most wetland plants (Caffrey and Kemp, 1991; Pedersen *et al.*, 1998; Lee and Dunton, 2000), therefore the findings from this study are important for both natural and constructed wetland ecosystems. This rhizosphere-driven anammox process was largely overlooked thus far. Taken together, we propose a conceptual model of N loss from paddy soil via different pathways along the redox gradient in paddy soils (Figure 4).

## Figures and Tables

**Table 1:** Characteristics of the paddy soil used in this study

*pH 1:2.5*	*SOM, g kg*^−*1*^	*Total N, g kg*^−*1*^	*C*_*mir*_*, mg kg*^−*1*^	*N*_*mic*_*, mg kg*^−*1*^	*Available P, mg kg*^−*1*^	*Available K, mg kg*^−*1*^	*Clay content %*
5.9±0.1	41.7±2.3	2.3±0.2	1092.6±21.4	128.2±3.2	9.5±0.8	62.8±5.6	13.2

Abbreviations: C_mic_, microbial biomass carbon; N_mic_, microbial biomass nitrogen; SOM, soil organic matter. Parent material: quaternary red clay. Mean±s.d. (*n*=3).

## References

[bib1] Armstrong W 1971 Radial oxygen losses from intact rice roots as affected by distance from the apex, respiration and waterlogging Physiol Plant 25 192 197

[bib2] Brune A Frenzel P Cypionka H 2000 Life at the oxic–anoxic interface: microbial activities and adaptations FEMS Microbiol Rev 24 691 710 11077159 10.1111/j.1574-6976.2000.tb00567.x

[bib3] Caffrey JM Kemp WM 1991 Seasonal and spatial patterns of oxygen production, respiration and root rhizome release in *Potamogeton perfoliatus* L and *Zostera marina* L Aquat Bot 40 109 128

[bib4] Dalsgaard T Canfield DE Petersen J Thamdrup B Acuña-González J 2003 N_2_ production by the anammox reaction in the anoxic water column of Golfo Dulce, Costa Rica Nature 422 606 608 12686998 10.1038/nature01526

[bib5] Fan G Zhu G Wang Y Wang S Wang C Yin C 2010 New functional microorganisms in nitrogen cycle restoration of river riparian ecosystems Acta Scien Circum 30 1558 1563

[bib6] Firestone MK Smith MS Firestone RB Tiedje JM 1979 Influence of nitrate, nitrite, and oxygen on the composition of the gaseous products of denitrification in soil Soil Sci Soc Am J 43 1140 1144

[bib7] Firestone MK Tiedje JM 1979 Temporal change in nitrous-oxide and dinitrogen from denitrification following onset of anaerobiosis Appl Environ Microbiol 38 673 679 16345447 10.1128/aem.38.4.673-679.1979 PMC243559

[bib8] Hamersley MR Lavik G Woebken D Rattray JE Lam P Hopmans EC 2007 Anaerobic ammonium oxidation in the Peruvian oxygen minimum zone Limnol Oceanogr 52 923 933

[bib9] Harhangi HR Le Roy M van Alen T Hu B Groen J Kartal B 2012 Hydrazine synthase, a unique phylomarker with which to study the presence and biodiversity of anammox bacteria Appl Environ Microbiol 78 752 758 22138989 10.1128/AEM.07113-11 PMC3264106

[bib10] Hefting M Beltman B Karssenberg D Rebel K van Riessen M Spijker M 2006 Water quality dynamics and hydrology in nitrate loaded riparian zones in the Netherlands Environ Pollut 139 143 156 15996804 10.1016/j.envpol.2005.04.023

[bib11] Huang Q Wang Q Luo Z Yu Y Jiang R Li H 2014 Effects of root iron plaque on selenite and selenate dynamics in rhizosphere and uptake by rice (*Oryza sativa* Plant Soil 1 12

[bib12] Humbert S Tarnawski S Fromin N Mallet M-P Aragno M Zopfi J 2010 Molecular detection of anammox bacteria in terrestrial ecosystems: distribution and diversity ISME J 4 450 454 20010634 10.1038/ismej.2009.125

[bib13] Humbert S Zopfi J Tarnawski SE 2012 Abundance of anammox bacteria in different wetland soils Environ Microbiol Rep 4 484 490

[bib14] Jackson LE Bowles TM Hodson AK Lazcano C 2012 Soil microbial-root and microbial-rhizosphere processes to increase nitrogen availability and retention in agroecosystems Curr Opin Env Sust 4 517 522

[bib15] Jetten MS Strous M Pas-Schoonen KT Schalk J Dongen UG Graaf AA 1998 The anaerobic oxidation of ammonium FEMS Microbiol Rev 22 421 437 9990725 10.1111/j.1574-6976.1998.tb00379.x

[bib16] Jia Y Huang H Zhong M Wang F-H Zhang L-M Zhu Y-G 2013 Microbial arsenic methylation in soil and rice rhizosphere Environ Sci Technol 47 3141 3148 23469919 10.1021/es303649v

[bib17] Jones D Hinsinger P 2008 The rhizosphere: complex by design Plant Soil 312 1 6

[bib18] Juretschko S Timmermann G Schmid M Schleifer KH Pommerening-Roser A Koops HP 1998 Combined molecular and conventional analyses of nitrifying bacterium diversity in activated sludge: *Nitrosococcus mobilis* and Nitrospira-like bacteria as dominant populations Appl Environ Microbiol 64 3042 3051 9687471 10.1128/aem.64.8.3042-3051.1998 PMC106813

[bib19] Kartal B Keltjens JT Jetten M 2008 The metabolism of anammox Encyclopedia of Life Sciences (ELS) John Wiley & Sons Ltd: Chichester, UK 1 9

[bib20] Kartal B Maalcke WJ de Almeida NM Cirpus I Gloerich J Geerts W 2011 Molecular mechanism of anaerobic ammonium oxidation Nature 479 127 130 21964329 10.1038/nature10453

[bib21] Kuypers MM Sliekers AO Lavik G Schmid M Jørgensen BB Kuenen JG 2003 Anaerobic ammonium oxidation by anammox bacteria in the Black Sea Nature 422 608 611 12686999 10.1038/nature01472

[bib22] Kuypers MM Lavik G Woebken D Schmid M Fuchs BM Amann R 2005 Massive nitrogen loss from the Benguela upwelling system through anaerobic ammonium oxidation Proc Natl Acad Sci USA 102 6478 6483 15843458 10.1073/pnas.0502088102 PMC556276

[bib23] Lam P Jensen MM Lavik G McGinnis DF Muller B Schubert CJ 2007 Linking crenarchaeal and bacterial nitrification to anammox in the Black Sea Proc Natl Acad Sci USA 104 7104 7109 17420469 10.1073/pnas.0611081104 PMC1849958

[bib24] Lee KS Dunton KH 2000 Diurnal changes in pore water sulfide concentrations in the seagrass Thalassia testudinum beds: the effects of seagrasses on sulfide dynamics J Exp Mar Biol Ecol 255 201 214 11108852 10.1016/s0022-0981(00)00300-2

[bib25] Li M Gu JD 2011 Advances in methods for detection of anaerobic ammonium oxidizing (anammox) bacteria Appl Microbiol Biotechnol 90 1241 1252 21476137 10.1007/s00253-011-3230-6 PMC3082692

[bib26] Liu D Fang S Tian Y Chang SX 2014 Nitrogen transformations in the rhizosphere of different tree types in a seasonally flooded soil Plant Soil Environ 60 249 254

[bib27] Liu WJ Zhu YG Hu Y Williams PN Gault AG Meharg AA 2006 Arsenic sequestration in iron plaque, its accumulation and speciation in mature rice plants (*Oryza sativa* L.) Environ Sci Technol 40 5730 5736 17007133 10.1021/es060800v

[bib28] Neef A Amann R Schlesner H Schleifer K-H 1998 Monitoring a widespread bacterial group: *in situ* detection of planctomycetes with 16S rRNA-targeted probes Microbiology 144 3257 3266 9884217 10.1099/00221287-144-12-3257

[bib29] Pedersen O Borum J Duarte CM Fortes MD 1998 Oxygen dynamics in the rhizosphere of *Cymodocea rotundata* Marine Ecol Progr Ser 169 283 288

[bib30] Richardson AE Barea J-M McNeill AM Prigent-Combaret C 2009 Acquisition of phosphorus and nitrogen in the rhizosphere and plant growth promotion by microorganisms Plant Soil 321 305 339

[bib31] Risgaard-Petersen N Meyer RL Schmid M Jetten MSM Enrich-Prast A Rysgaard S 2004 Anaerobic ammonium oxidation in an estuarine sediment Aquat Microb Ecol 36 293 304

[bib32] Sato Y Ohta H Yamagishi T Guo Y Nishizawa T Rahman MH 2012 Detection of anammox activity and 16S rRNA genes in ravine paddy field soil Microbes Environ 27 316 319 22353769 10.1264/jsme2.ME11330 PMC4036040

[bib33] Schmid M Twachtmann U Klein M Strous M Juretschko S Jetten M 2000 Molecular evidence for genus level diversity of bacteria capable of catalyzing anaerobic ammonium oxidation Syst Appl Microbiol 23 93 106 10879983 10.1016/S0723-2020(00)80050-8

[bib34] Schmid MC Maas B Dapena A de Pas-Schoonen KV de Vossenberg JV Kartal B 2005 Biomarkers for *in situ* detection of anaerobic ammonium-oxidizing (anammox) bacteria Appl Environ Microbiol 71 1677 1684 15811989 10.1128/AEM.71.4.1677-1684.2005 PMC1082507

[bib35] Shen L Liu S Lou L Liu W Xu X Zheng P 2013 Broad distribution of diverse anaerobic ammonium-oxidizing bacteria in Chinese agricultural soils Appl Environ Microbiol 79 6167 6172 23747706 10.1128/AEM.00884-13 PMC3811364

[bib36] Sliekers AO Derwort N Campos-Gomez JL Strous M Kuenen JG Jetten MSM 2002 Completely autotrophic nitrogen removal over nitrite in one single reactor Water Res 36 2475 2482 12153013 10.1016/s0043-1354(01)00476-6

[bib37] Steen E 1984 Variation of root-growth in a grass ley studied with a mesh bag technique Swed J Agric Res 14 93 97

[bib38] Steen E Atkinson D 1991 Usefulness of the mesh bag method in quantitative root studies Plant Root Growth: An Ecological Perspective Blackwell scientific publications LTD: Oxford, England, UK 75 86

[bib39] Strous M Heijnen JJ Kuenen JG Jetten MSM 1998 The sequencing batch reactor as a powerful tool for the study of slowly growing anaerobic ammonium-oxidizing microorganisms Appl Microbiol Biotechnol 50 589 596

[bib40] Thamdrup B Dalsgaard T 2002 Production of N_2_ through anaerobic ammonium oxidation coupled to nitrate reduction in marine sediments Appl Environ Microbiol 68 1312 1318 11872482 10.1128/AEM.68.3.1312-1318.2002 PMC123779

[bib41] Wallner G Amann R Beisker W 1993 Optimizing fluorescent *in situ* hybridization with rRNA-targeted oligonucleotide probes for flow cytometric identification of microorganisms Cytometry 14 136 143 7679962 10.1002/cyto.990140205

[bib42] Wang J Gu J 2013 Dominance of *Candidatus Scalindua* species in anammox community revealed in soils with different duration of rice paddy cultivation in Northeast China Appl Microbiol Biotechnol 97 1785 1798 22526793 10.1007/s00253-012-4036-x PMC3562551

[bib43] Wang S Zhu G Peng Y Jetten MS Yin C 2012 Anammox bacterial abundance, activity, and contribution in riparian sediments of the Pearl River estuary Environ Sci Technol 46 8834 8842 22816681 10.1021/es3017446

[bib44] Wang Y Zhu G Harhangi HR Zhu B Jetten MSM Yin C 2012 Co-occurrence and distribution of nitrite-dependent anaerobic ammonium and methane-oxidizing bacteria in a paddy soil FEMS Microbiol Lett 336 79 88 22889245 10.1111/j.1574-6968.2012.02654.x

[bib45] Xing GX Zhu ZL 2000 An assessment of N loss from agricultural fields to the environment in China Nutr Cycl Agroecosys 57 67 73

[bib46] Zhu G Jetten MS Kuschk P Ettwig KF Yin C 2010 Potential roles of anaerobic ammonium and methane oxidation in the nitrogen cycle of wetland ecosystems Appl Microbiol Biotechnol 86 1043 1055 20195861 10.1007/s00253-010-2451-4

[bib47] Zhu G Wang S Feng X Fan G Jetten MS Yin C 2011 Anammox bacterial abundance, biodiversity and activity in a constructed wetland Environ Sci Technol 45 9951 9958 21981702 10.1021/es202183w

[bib48] Zhu G Wang S Wang Y Wang C Risgaard-Petersen N Jetten MSM 2011 Anaerobic ammonia oxidation in a fertilized paddy soil ISME J 5 1905 1912 21593796 10.1038/ismej.2011.63 PMC3223303

[bib49] Zhu G Wang S Wang W Wang Y Zhou L Jiang B 2013 Hotspots of anaerobic ammonium oxidation at land-freshwater interfaces Nat Geosci 6 103 107

[bib50] Zhu Z 2008 Research on soil nitrogen in China Acta Pedologica Sin 45 778 783

