# Supplementary material for: Nitrogen loss by anaerobic oxidation of ammonium in rice rhizosphere
Source: ISME J. 2015 Feb 17;9(9):2059–67. doi: 10.1038/ismej.2015.25 (PMC4542037; doi:10.1038/ismej.2015.25)
Supplement: Supplementary Information [file ismej201525x1.doc]

# Nitrogen loss by anaerobic ammonium oxidation in rice rhizosphere

San’an Nie^1^, Hu Li^1^, Xiaoru Yang^1^, ZhaoJi Zhang^1^, Bosen Weng^1^, Fuyi Huang^1^, Guibing Zhu^*2^, Yong-Guan Zhu^*1^

1. Key Laboratory of Urban Environment and Health, Institute of Urban Environment, Chinese Academy of Sciences, Xiamen 361021, China
2. Key Laboratory of Drinking Water Science and Technology, Research Center for Eco-Environmental Sciences, Chinese Academy of Sciences, Beijing 100085, China

Table S1. Basic proposed of CARD-FISH protocol for visualization of ammonia oxidizing bacteria genera ‘*Ca* Brocadia’ and ‘Ca. Kuenenia’ in the soils

| Stage | Step | Description |
| --- | --- | --- |
| *In* *situ* cells fixation and dehydration | 1 | About 1.0 g of raw soil were add with 2 mL PBS (0.2 M) buffer and then ultrasound dispersed at minimum power for 60 s. |
|  | 2 | Add formaldehyde to the cell suspension to a final concentration of 4% (v/v). Fix the sample for 12 h at 4^o^C. Then centrifuge the fixed sample at 10,000 × g for 5 minutes, discard 2ml of the supernatant and add 2 mL of 1 × PBS. Repeat twice and stored in ethanol solution (50%) at -20^o^C. |
|  | 3 | Add 900 μL of 1 × PBS to 100 μL of the fixed sample then sonication with a sonication probe at minimum power for 20s. |
|  | 4 | Select 10 μL of the above solution on a gelatin embedded slides. Dehydrate the slides in 50%, 80% and 99.5% ethanol solution. Then dry the slides at room temperature. |
| Inactivation of endogenous peroxidases | 5 | Incubate the slides in 50 mL of H_2_O_2_ (0.1%) for 2 minutes at room temperature. Wash the slides and stored them at 4^o^C. |
| Permeabilization | 6 | Incubate the slides in 10 mg mL^-1^ lysozyme (Roche, Mannheim, Germany) solution at 37^o^C for 1 h. Wash the slides and dry the slides at room temperature. |
| *In* *situ* hybridization | 7 | Dilute the HRP-conjugated oligonucleotide probe (Amx820) with hybridization buffer. Add 10 μL of probe hybridization mix onto the slides, hybridize the samples in a humidified chamber for 2 h at 46°C. |
|  | 8 | Wash the slides in 50 mL clearing solution for 20 minutes at 46°C. |
| Catalyzed reporter deposition | 9 | Wash the slides in 50 mL 1 × PBS for 15 minutes at room temperature.  Mixture solution preparation: add 1ml of amplification buffer with 10 μL of H_2_O_2_ (0.01%) and 2 μL of fluorescently labeled tyrosine (TSA™ Reagent, Alexa Fluor® 568 Tyramide, Molecular Probes, Inc. Eugene, OR). |
|  | 10 | Add 10 μL of above solution onto the slides surface and incubate in the darkness for 15 minutes at 46°C. Wash the samples with 1 × PBS twice for 15 minutes. Then, wash the samples with 50% ethanol for 1 min and air dry. |
|  | 12 | Record the fluorescence signals with an LSM 710 confocal laser scanning microscope (CLSM) (Carl Zeiss, Inc., Germany) and analyze the pictures by the standard software for the LSM 710. |

Table S2. Isotope tracing technique equations used for the calculation of anammox and denitrification activity, and their contributions to N_2_ production

| Equation | Explanation | # |
| --- | --- | --- |
| $A_{28}=A_{total}\times(1-F_{N})$ | Anammox using F_N_ and A_total_ | 1 |
| $A_{29}=A_{total}\times F_{N}$ |  | 2 |
| $A_{total}=\mathrm{F}_{N}^{-1}\times[P_{29}+2\times(1-F_{N}^{-1})\times\mathrm{P}_{30}]$ | Anammox using F_N_, P_29_ and P_30_ | 3 |
| $D_{28}=D_{total}\times(1-\mathrm{F}_{N})^{2}$ | Denitrification using F_N_ and D_total_ | 4 |
| $D_{29}=D_{total}\times2\times(1-F_{N})\times\mathrm{F}_{N}$ |  | 5 |
| $D_{30}=D_{total}\times\mathrm{F}_{N}^{2}$ |  | 6 |
| $D_{total}=P_{30}\times\mathrm{F}_{N}^{-2}$ | Denitrification using F_N_ and P_30_ | 7 |
| $R_{d}\%=\frac{D_{total}}{D_{total}+A_{total}}\times100\%$ | Proportion of denitrification in the total N_2_ production | 8 |
| $R_{a}\%=\frac{A_{total}}{D_{total}+A_{total}}\times100\%$ | Proportion of anammox in the total N_2_ production | 9 |

Where F_N_ is the fraction of ^15^N in NO_3_^-^ (98.15%), P_29_ and P_30_ representing the measured production of ^29^N_2_ and ^30^N_2_ in the ^15^NO_3_^-^ treatment, D_m_ and A_m_ denote production of N_2_ by anammox and denitrification, respectively. A_total_ = A_28_ + A_29_, D_total_ = D_28_ + D_29_ + D_30_. R_d_% and R_a_% representing the contribution of denitrification and anammox to total N_2_ production, respectively.


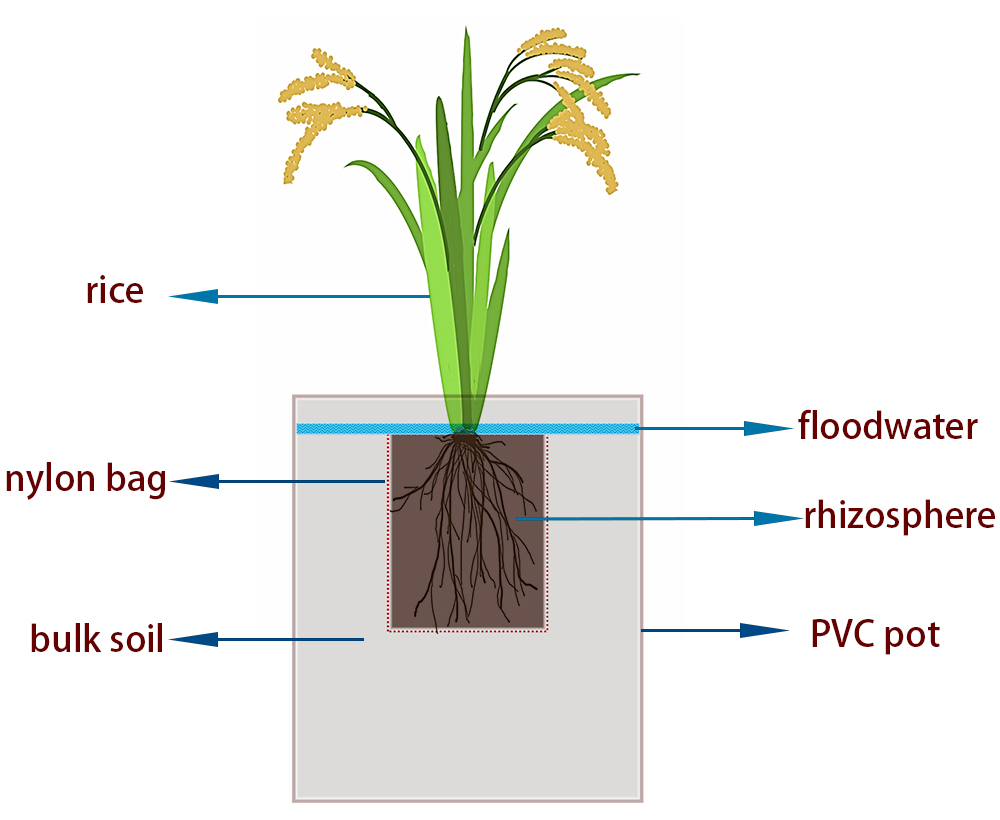


Figure S1. The rhizo-bag and pot experiment setup for rice growth

Figure S2 The NO_x_^-^ concentration in the pre-incubation experiment

The soil samples evaluated were (1) rhizosphere in control (RC); (2) non-rhizosphere in control (NC); (3) rhizosphere in N fertilization (RN); (4) non-rhizosphere in N fertilization (NN).

Figure S3 Example of concentrations of ^29^N_2_ and ^30^N_2_ in samples amended with ^15^N labeled on ammonium and nitrate separately
